# The Effect of Antipsychotics on Cognition in Schizophrenia—A Current Narrative Review

**DOI:** 10.3390/brainsci14040359

**Published:** 2024-04-03

**Authors:** Petru Fabian Lungu, Corina Miruna Lungu, Alin Ciobica, Ioana Miruna Balmus, Raluca Vitalaru, Ioannis Mavroudis, Romeo Dobrin, Mirela Cimpeanu, Irina Luciana Gurzu

**Affiliations:** 1 Faculty of Biology, Biology Department, “Alexandru Ioan Cuza” University, 700506 Iasi, Romania; lungufabian123@gmail.com (P.F.L.); mirela.cimpeanu@uaic.ro (M.C.); 2 Faculty of Psychology and Educational Sciences, Psychology Department, “Alexandru Ioan Cuza” University, 700506 Iasi, Romania; 3 Center of Biomedical Research, Romanian Academy, Iasi Branch, Teodor Codrescu 2, 700481 Iasi, Romania; 4 Academy of Romanian Scientists, 3 Ilfov, 050044 Bucharest, Romania; 5 Preclinical Department, Apollonia University, Strada Păcurari 11, 700511 Iasi, Romania; 6 Institute of Interdisciplinary Research, Department of Exact Sciences and Natural Sciences, “Alexandru Ioan Cuza” University, 700506 Iasi, Romania; balmus.ioanamiruna@yahoo.com; 7 CENEMED Platform for Interdisciplinary Research, “Grigore T. Popa” University of Medicine and Pharmacy of Iasi, 16th Universitatii Street, 700115 Iasi, Romania; 8 Institute of Psychiatry “Socola”, Iasi Str. Bucium 36, 700282 Iasi, Romaniaromeodobrin2002@gmail.com (R.D.); 9 Department of Neuroscience, Leeds Teaching Hospitals, NHS Trust, Leeds LS2 9JT, UK; 10 Faculty of Medicine, “Grigore T. Popa” University of Medicine and Pharmacy of Iasi, 700115 Iasi, Romania; irina-luciana.gurzu@umfiasi.ro

**Keywords:** schizophrenia, antipsychotics, cognition

## Abstract

The majority of schizophrenia-affected individuals display deficiencies in multiple cognitive domains such as attention, working memory, long-term memory, and learning, deficiencies that are stable throughout the disease. The purpose of this narrative review was to examine the effect of antipsychotics on several cognitive domains affected by schizophrenia. **Methods:** We searched MEDLINE, Elsevier, Scopus, and DOAJ databases for randomized controlled trials and other studies investigating the effects of typical and atypical antipsychotics on cognition in patients with schizophrenia in studies conducted in the last decade. **Results:** The majority of studies included in this review showed that antipsychotics (especially SGAs) have positive effects on both cognition and general psychopathology of schizophrenia. We mention that treatment with antipsychotic substances represents an ongoing effort of the researchers, who are constantly searching for the best approach to meet the mental health needs of schizophrenia patients. **Conclusions:** Even with those positive results, it should be noted that more studies are needed in order to fully observe the various effects of certain antipsychotic substances on cognition.

## 1. Introduction

Schizophrenia spectrum disorders are defined by fundamental and characteristic cognitive and perceptual distortions as well as affective flattening, avolition, and social withdrawal. Although certain cognitive deficiencies may develop over time, clear consciousness and intellectual abilities are usually preserved [1]. Paranoid schizophrenia and schizoaffective disorders are the most studied of all schizophrenia spectrum disorders, although hebephrenic schizophrenia is more prevalent (0.32% for both paranoid schizophrenia and schizoaffective disorders, compared to 1% for hebephrenia) [2,3].

Paranoid schizophrenia represents a serious mental disorder with a prevalence of 58.6% in schizophrenia individuals, and it is defined by symptoms such as hallucinations (mainly auditory), disorganized thinking, and delusions. Other symptoms include apathy, affective flattening, and social withdrawal. Symptoms usually begin in early adulthood, develop gradually, and in many cases do not resolve completely [1,4]

Hebephrenic schizophrenia (also known as disorganized schizophrenia) is a specific form of schizophrenia in which emotional changes are predominant. The clinical picture of this psychiatric condition also includes ephemeral and fragmentary delusions and hallucinations, unpredictable behavior, disorganized thought and speech, shallow mood, and common mannerisms. Hebephrenia should be a diagnosis reserved only for adolescents or young adults [1].

Schizophrenia affects nearly 24 million people or 1 in 300 people worldwide, according to the World Health Organization. Research has not yet found a specific cause of schizophrenia, although a series of factors of an environmental, genetic, or chemical nature can play an essential role in its occurrence and development. For example, the season of birth (particularly late winter/early spring), late age of father, pregnancy and birth complications, stress, malnutrition, certain infections, and maternal diabetes have all been connected to a higher risk for developing schizophrenia [5].

In the clinical evaluation of schizophrenia, positive symptoms, negative symptoms, and general psychopathology must be taken into account.

Positive symptoms are usually represented by the following:

- hallucinations (most commonly auditory);
- disorganized thought and speech;
- psychomotor agitation and delusional ideas.

Negative symptoms are represented in particular by the following:

- affective flattening;
- social withdrawal;
- stereotypic thinking;
- anhedonia, avolition, and abstract thinking difficulties.

In terms of general psychopathology, clinicians and psychiatrists must look for symptoms such as somatic preoccupation, anxiety, depression, ideas of guilt, as well as cognitive deficits (including but not limited to unusual thought content, disorientation, attention deficit, poor control of impulses, and anosognozia) [5].

The first-line treatment for schizophrenia is represented by antipsychotics. Antipsychotic drugs are often divided into two distinct categories and are used to treat and manage symptoms for a variety of psychiatric conditions. First-generation antipsychotics, or FGAs, commonly referred to as “typical antipsychotics”, were first developed in the 1950s. SGAs, or “atypical antipsychotics”, or second-generation antipsychotics, became available in the 1980s. Regarding their mechanism of action, first-generation antipsychotics work by inhibiting dopaminergic neurotransmission (with the mention that they are the most effective when they block approximately 72% of the brain’s D2 dopamine receptors) as well as cholinergic, noradrenergic, and histaminergic activity, whereas second-generation antipsychotics work by blocking D2 dopamine receptors as well as serotonin receptor antagonist action, the most commonly involved being the 5-HT2A subtype [6].

Although atypical antipsychotics (including paliperidone, ziprasidone, and lurasidone) have shown certain cognitive benefits, high antipsychotic dosages are linked to an array of cognitive deficits in schizophrenia patients [7,8,9,10].

There is evidence that first-generation antipsychotic medication generates extrapyramidal side effects, such as akathisia, dystonia, parkinsonism, and tardive diskinezia [11,12]. Despite the fact that second-generation (SGA) antipsychotics have increasingly displaced first-generation (FGA) antipsychotics in industrialized countries, the former are still the most often prescribed drugs in many other regions of the world, particularly poor countries, since they are significantly less expensive [11,12].

Cannabidiol is a non-psychoactive substance that has been associated with antipsychotic and anxiolytic effects. It may also have neuroprotective effects on the human brain. It seems to be a potential adjuvant treatment for neurodegenerative diseases, but its effectiveness in improving cognitive functions in schizophrenia is arguable [13,14,15,16].

Cannabidiol (CBD) first demonstrated its therapeutic effectiveness in a patient who did not respond to haloperidol treatment, and later for a patient with Parkinson’s disease who exhibited psychotic symptoms. Compared to amisulpride, CBD demonstrated similar effectiveness in relieving those symptoms, and only a few adverse effects were reported [17,18,19].

In the following paragraphs, we will go into detail about all the antipsychotics that were investigated in the studies that were included in this review, following a series of general characteristics.

### 1.1. Clozapine

Clozapine is used for the treatment of schizophrenia patients who do not respond to other antipsychotic treatments [20]. This characteristic of clozapine is also shared by first-generation antipsychotics [21]. The main issue is that its efficacy in improving cognition as well as its safety is not yet clear [22,23,24].

### 1.2. Blonaserin

Blonaserin is a new antipsychotic drug that was firstly distributed in Japan in 2008, followed by Korea, then China in 2017, for the treatment of schizophrenia. Blonaserin has high selectivity for D2 receptors and serotonin 5-HT24, as well as for D3 receptors and low affinity for D1 receptors, serotonin 5-HT2C, adrenaline alpha 1, histamine H1, and muscarinic M1 receptors [25].

### 1.3. Lurasidone

Lurasidone is a commonly used atypical antipsychotic that is very similar to clozapine and has a stronger 5-HT2A serotonin receptor antagonist effect than dopamine D2. The mechanism that gives this antipsychotic its efficacy is the inhibition of 5-HT1A (partial) and 5-HT7 receptors. In preclinical studies, lurasidone was found to be an effective treatment for psychotic episodes and cognitive deficits [26,27].

### 1.4. Risperidone

Risperidone is an atypical antipsychotic medication used to treat psychotic disorders. It is a benzisoxazole derivate according to its molecular structure and a monoaminergic antagonist with a strong affinity for serotonergic and dopaminergic receptors. Risperidone binds to alpha 1-adrenergic receptors, as well as histaminergic and 2-adrenergic receptors to a lesser extent, but it does not bind to cholinergic receptors. Risperidone comes in a variety of forms (oral tablets, orally disintegrating tablets, oral solution, or long-acting intramuscular injection) which are administered in different concentrations (0.5 mg, 1 mg, 2 mg, 3 mg, 4 mg, and 6 mg). Risperidone is also the first SGA to come in an injectable form [28].

### 1.5. Ziprasidone

Ziprasidone is an atypical antipsychotic with a low-to-medium affinity for norepinephrine transporter (NET) and serotonin transporter (SERT) which are transport pathways for antidepressant medication. In addition to ziprasidone, it has increased affinity for multiple serotonergic receptors such as 5-HT1A, 5-HT2A, 5-HT2C, and 5-HT7. It is worth mentioning that all these receptors can be used for the development of new antidepressants. Current studies suggest that ziprasidone may be a very good mood stabilizer [29,30,31].

## 2. Aim

Schizophrenia remains a serious psychiatric condition that influences many aspects of the patient’s life, one of them (and the most important) being cognitive functioning. Schizophrenia symptomatology can be managed with the aid of antipsychotic agents, which can have a significant impact on the patient’s cognitive functioning. However, little is known about how antipsychotic drugs can affect certain cognitive domains and processes (such as working memory, attention, learning, etc.). More studies are needed in order to determine the specific mechanism of action for each antipsychotic substance.

Our narrative review aims to group in one place the unique contributions that each of the included studies have in determining the effect of antipsychotics on cognitive functions, symptomatology, and general psychopathology for people affected by schizophrenia.

## 3. Method

The sources consulted for this article were electronic databases such as MEDLINE, Elsevier, Scopus, and DOAJ. The initial search strategy involved the use of the following thesaurus terms: ‘“olanzapine treatment” schizophrenia’; ‘olanzapine treatment in schizophrenia’; ‘lurasidone treatment in schizophrenia’; ‘lurasidone in schizophrenia’; ‘blonanserin in schizophrenia’; ‘cannabidiol treatment in schizophrenia’; ‘risperidone in schizophrenia’; ‘second generation antipsychotics effectiveness in schizophrenia’; ‘ziprasidone in schizophrenia’; ‘antipsychotics effectiveness for acute schizophrenia’; ‘clozapine treatment for improving cognitive performance in schizophrenia’; ‘amisulpride treatment for improving cognitive performance in schizophrenia’; ‘paliperidone treatment for cognitive improvement in schizophrenia patients’. It is important to mention that the last literature search was on 8 September 2023.

We included studies with the following criteria:

- Were first released in English. Abstracts and titles were examined for the initial inclusion of studies. If the abstract’s content was promising, the complete text was carefully examined to establish the aim, method (including instruments, participants, and procedure), and final results.
- Used antipsychotics as the primary treatment and examined their effect on cognition and symptomatology.
- Included only participants diagnosed with a schizophrenia spectrum disorder.
- Had substantial cohorts (except for pilot studies or case–control studies).
- Had different study designs (intervention, pilot, random trials, observational, etc.).
- Presented at least one favorable cognitive outcome for the antipsychotic agent/agents used.
- Had mostly human participants, regardless of whether they were adult or juvenile.

We excluded studies with the following criteria:

- Used antipsychotics in the testing phase.
- Mostly used animals as subjects.
- Were reviews and meta-analyses regarding this topic.
- Did not describe in detail the cognitive domains that were assessed when administering certain antipsychotic agents.
- Were not scientific articles.

**Figure 1:**
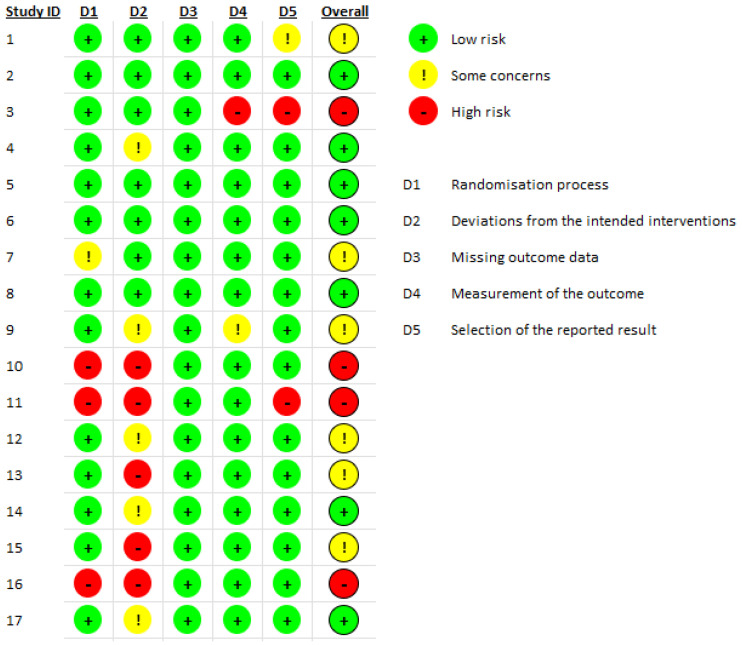
Risk of bias for the included studies.

After selecting the articles from the aforementioned database searches, we entered the articles into the Revised Cochrane risk of bias tool for randomized trials (RoB2) to determine the risk of bias, shown in the following flow diagram (Figure 1) [32].

## 4. Results

The following table summarizes methodological aspects such as the objectives and the number of participants from the studies included in this narrative review (Table 1). In the upcoming paragraphs, we described in greater detail the results presented in the included articles. There is also another table that contains the instruments used for assessment as well as the results for each study (Table 2).

### 4.1. Paliperidone

Li et al. expected social cognition to mediate the relationship between cognition and negative symptoms. Specifically, the researchers expected Social Cognition and Interaction Training (SCIT, a well-known therapeutic program) combined with paliperidone to be more effective than paliperidone alone in improving cognitive functioning and psychotic symptoms present in the early stages of schizophrenia [45,48].

The researchers divided patients into two different treatment groups, namely a group that received both SCIT and paliperidone (n = 106) and a group that received only paliperidone (n = 102). Those groups consisted of 5–8 patients/group and were led by trained-by-book clinicians, who learned the intervention from its creator. The sessions lasted between 45 and 60 min once a week for 20–24 weeks [45]. The instruments used for evaluating the patients’ cognitive functioning and symptomatology were the following: MATRICS Consensus Cognitive Battery (MCCB) [24], Scale for the Assessment of Negative Symptoms (SANS) [49], Positive and Negative Syndrome Scale (PANSS) [50], and Personal and Social Performance Scale (PSP) [51].

For the inclusion in the study, patients had to meet the following criteria: a schizophrenia diagnosis according to ICD-10, absence of cranial trauma, right-hand dexterity with good eyesight and unaffected hearing, no history of antipsychotic treatment or less than 2 weeks from the last treatment, and no long-acting injection of antipsychotic agents. The exclusion criteria included depression and suicidal thoughts, the presence of a severe or unstable physical disorder, substance or alcohol abuse 6 months prior to the study, and an IQ lower than 70 [34]. The results showed that SCIT provided significant added benefits beyond paliperidone in terms of processing speed, attention, and social cognition [45,52]. However, the combination of SCIT and paliperidone had no significant impact on social functioning or symptomatology for juvenile (young) patients with schizophrenia.

### 4.2. Amisulpride

Zhu et al. aimed to investigate the efficiency and safety of using amisulpride, in the case of CTRS (clozapine-resistant treatment-refractory schizophrenia) patients who received at least two doses of antipsychotics with different chemical structures over a long period and recently received a dose of clozapine (400 mg/day) for 6 months [39]. The study had as its main aim to investigate whether amisulpride improves general psychopathology and cognitive abilities [39].

Zhu et al. included a total number of 80 participants who were divided into two groups, 40 participants receiving a treatment consisting of amisulpride and clozapine, and the second group also numbering 40 were treated with clozapine and a placebo. Inclusion criteria were the following: Chinese ethnicity, age between 18 and 65 years, meets DSM-IV criteria for schizophrenia, received two different antipsychotics with different mechanism of action, possible resistance to treatment, and a score on the PANSS scale above 60. Exclusion criteria were the following: the presence of one of the other major Axis I disorders, a serious physical disorder, substance abuse, and pregnancy [39]. Instruments used in the study were the Positive and Negative Syndrome Scale (PANSS) [50], Repeatable Battery for the Assessment of Neuropsychological Status (RBANS) [53], Clinical Global Impressions Scale (CGI-S) [54], and the Treatment Emergent Symptoms Scale (TESS) [55]. The results demonstrated that clinical symptomatology and cognitive functioning of CTRS patients can be safely improved through amisulpride augmentation therapy. Moreover, when compared to the placebo group, CTRS patients in the amisulpride augmentation group showed improvement in both positive and general psychopathology at week 6, as well as week 12.

In a blind, semi-randomized study conducted by Johnsen et al., the researchers wanted to compare amisulpride, aripiprazole, and olanzapine in terms of their effectiveness in the treatment of patients with schizophrenia spectrum disorders [40]. The number of participants evaluated in the study was initially 359, of whom 215 were excluded (107 did not meet the eligibility criteria, 82 refused to participate, and 26 for other reasons). The remaining 144 participants (of which 51 were women and 93 were men) were at least 18 years old; were mostly Caucasian; had a PANSS score of ≥4 on P1 (delirium) items, P3 (hallucinations), P5 (grandiosity), P6 (suspicion or persecution), and G9 (unusual thought content); and had a diagnosis of a schizophrenia spectrum disorder according to DSM-IV criteria (axis 1) later converted into ICD-10 diagnoses of either paranoid schizophrenia (N = 84), schizotypal disorder (N = 2), persistent delusional disorder (N = 21), acute and transient psychotic disorders (N = 18), schizoaffective disorders (N = 10), nonorganic psychotic disorder (N = 1), or unspecified psychotic disorder (N = 8). They were randomly distributed in the three groups (amisulpride—44 patients; apiprazole—48 patients; and olanzapine—52 patients) [40].

The exclusion criteria were the following: the lack of understanding German or Norwegian language, pregnancy, breastfeeding, allergy to the active substances/excipients used in the study, the presence of prolactin-dependent tumors, phaeochromocytoma, concomitant use of drugs that could induce torsades de pointes (heart disease), levodopa use, and high risk of narrow-angle glaucoma [40]. The instruments used for assessing antipsychotics’ effectiveness were the Positive and Negative Syndrome Scale (PANSS) [50]; Structured Clinical Interview for PANSS [56]; Clinical Global Impressions Scale (CGI-S) [54]; and the Global Assessment of Functioning (GAF) [57]. To assess the safety of antipsychotics, the UKU-Side Effect Rating Scale, the patient-rated version, was used [40,58]. The results demonstrated that amisulpride was more effective than olanzapine, as indicated by a significantly reduced PANSS score on the positive symptoms subscale, but not significantly greater than aripiprazole. On the negative symptoms scale, however, no significant differences were found between antipsychotics. Overall, amisulpride had higher effectiveness than aripiprazole or olanzapine in lowering the PANSS total scores for adults with schizophrenia spectrum disorders. Very few adverse effects were reported and were common to all three antipsychotics [40].

### 4.3. Effects of Cannabidiol in Combination with Amisulpride

Another similar study carried out by Leweke and collaborators aimed to compare the effectiveness of monotherapeutic cannabidiol and an atypical antipsychotic amisulpride in the early phases of schizophrenia, for six neurocognitive domains [18]. Participants were aged between 18 and 50 years, had a diagnosis of either schizophrenia or schizophreniform psychosis, had a Brief Psychiatric Rating Scale (BPRS) score ≥36, and a BPRS thought disorder score ≥12. The standard procedure included a screening period that lasted 7 days and a minimum of 3 days of antipsychotic treatment discontinuation. Patients were excluded if they had a positive urine drug screening for cannabinoids or other illicit substances, other psychiatric disorders or relevant medical conditions, treatment resistance, or treatment with a depot antipsychotic three months prior to the study.

Of the 198 patients screened, only 42 were eligible for the trial. They were divided into two groups: one comprised of 21 schizophrenia or schizophreniform patients which received combined cannabidiol and amisulpride treatment and one consisting of 21 control participants who received a placebo, over the course of 4 weeks. The assessment of neurocognitive capacities lasted two hours for each participant and was carried out by experienced neurophysiologists [18]. A set of tests were used to assess different cognitive functions: pattern recognition was assessed with a computer version of the backward masking task using the letters F, H, and T as stimuli [18]; attention was measured with the Continuous Performance Test (CPT—identical pairs version) [59]; working memory was assessed using the Letter–Number Sequencing task (LNS) [60], together with the Subject Ordered Pointing Task (SOPT) [61]; spatial working memory was examined using the Delayed Response Task (DRT) [62,63]; and verbal and visual memory and learning were assessed using the Auditory Verbal Learning Test (AVLT) [64]. Visual memory was measured using the Rey-Osterricht Complex Figure Test (ROCF) [65]; processing speed was evaluated using the Digit Symbol Test (DST) [66] and Trail Making Test A and B (TMT) [67]; and verbal executive functions were evaluated with a verbal fluency task [68,69].

The findings indicate that both cannabidiol and amisulpride have comparable effects on neurocognitive functioning in young, acutely ill schizophrenia patients. Amisulpride increased the functioning of both working memory and visual memory [18], while cannabidiol treatment led to an improvement in working and visual memory performance, as well as processing speed, visuomotor coordination, visual memory, and sustained attention. However, both treatment groups had similar effect sizes [18].

### 4.4. Clozapine and Quetiapine

Park et al. aimed to observe the association between clozapine N-desmethylclozapine (NDMC, which is a major active metabolite of clozapine) and the improvement in cognitive functioning in schizophrenia patients. This was a retrospective study that took place between 2016 and 2019. Patients were included if they had a diagnosis of schizophrenia or a schizoaffective disorder according to ICD-10 criteria, were 19 or older, were in a post-acute phase, and had 2 months of stable treatment; only 15 patients (mostly female) met the eligibility criteria. No exclusion criteria were mentioned in the study. Blood was collected from schizophrenia patients in order to determine the level of clozapine and N-desmethylclozapine, using the mass spectrophotometry method by tandem liquid chromatography [43,70].

The clinical and cognitive assessment was performed with the MATRICS Consensus Cognitive Battery (MCCB) [24] which is comprised of 10 tests for evaluating seven cognitive domains (processing speed, attention/vigilance, working memory, verbal learning, visual learning, reasoning/problem solving, and social cognition). The Continuous Performance Test–Identical Pairs (CPT-IP) [59] was used for assessing processing speed; the Spatial Span test (SS) and Letter–Number Span test (LNS) of the Weschler Memory Scale 3rd edition (WMS-III) [71] were used for assessing working memory; the Hopkins Verbal Learning Test-Revised (HVLT-R) was used for assessing verbal learning; the Brief Visuospatial Memory Test-Revised (BVMT-R) was used for assessing visual learning [72]; the NAB-Mazes Test was used for assessing capacity for reasoning and problem solving [73]; the Managing Emotions subscale (ME) of the Mayer–Salovey–Caruso Emotional Intelligence Test (MSCEIT) was used [74]. The results showed that attention/vigilance and social cognition were significantly and negatively correlated with the clozapine/NDMC ratio, while working memory had a negative correlation with both clozapine concentration and NDMC concentration. Moreover, social cognition was inversely correlated with clozapine concentration. Overall, clozapine improved cognitive functions (especially attention but also working memory and social cognition, depending on the dose administered) [43].

In a follow-up study by Hui et al., conducted between 2003 and 2014, the researchers wanted to see if discontinuing antipsychotic treatment had long-term consequences [44]. There were 178 participants monitored by Hui et al., of which 80 were men and 98 were women, aged between 18 and 65 years, who received constant medication for 12 months, and no longer had positive symptoms or relapses (an increase in the frequency of positive symptoms requiring hospitalization). Their diagnosis was established using the Chinese version of the Structured Clinical Interview for DSM-IV. The exclusion criteria were the following: current treatment with clozapine depot or mood stabilizers, low adherence to treatment, suicidal tendencies, or aggressive behavior.

Patients were randomly assigned to two equal groups: early discontinuation of treatment or maintenance treatment (oral quetiapine 400 mg daily), for 12 months [44]. The instruments used for the assessment of patients were the Positive and Negative Syndrome Scale (PANSS) [50], Clinical Global Impressions Scale (CGI) [54], Calgary Depression Scale for Schizophrenia (CDSS) [75], Scale to Assess Unawareness of Mental Disorder (SUMD) [76], Short Form Survey-36 [77], Simpson–Angus Scale (SAS) [78], Abnormal Involuntary Movement Scale (AIMS) [79], Barnes Akathisia Rating Scale (BARS) [80], UKU Side Effects Rating Scale [58], and UKU Global Side Effects [44].

The results indicated that 35 (39%) of 89 patients in the discontinuance group and 19 (21%) of 89 patients in the maintenance therapy group had a poor 10-year clinical outcome (risk ratio 1.84, 95% CI 1.15–2.96; *p* = 0.012). In the follow-up phase, suicide was the only significant adverse event, with 4% of the participants in the early discontinuation group and 2% in the maintenance group [44].

### 4.5. Ziprasidone

Stip and his team aimed to determine whether ziprasidone administered to patients leads to functional and structural changes in the brain and in the regions responsible for cognition and emotion [47].

There were 30 participants in the study with schizophrenia with recent psychotic episodes (last 5 years) and 15 healthy controls. Half of the participants with schizophrenia received ziprasidone upon enrollment in the study and the rest of them received different antipsychotic agents such as quetiapine (n = 4), risperidone (n = 2), olanzapine (n = 4), perphenazine (n = 1), and a combination of clozapine and quetiapine (n = 1). Patients were included if they were aged between 18 and 40 years, had a recent diagnosis of schizophrenia (last 5 years), and were able to undergo a MRI scan without needing anxiolytics. The exclusion criteria were the following: the presence of other neurological disorders, changes in the treatment, substance abuse, and abnormalities clearly visible on the MRI scan [47].

For the psychiatric and neuropsychological assessment, the following instruments were used: Positive and Negative Syndrome Scale (PANSS) [50] for evaluating the positive and negative symptoms, Calgary Depression Scale for Schizophrenia (CDSS) [75] for determining the severity of depression, Barratt Impulsiveness Scale (BIS) [81] to determine the degree of impulsivity, and Edinburgh Handedness Inventory (EHI) [82] to determine hand dominance. The cognition–emotion-type task consisted of two neuroimaging sessions, one at the beginning and one at the end, and brain activity was measured (using the blood-oxygenation-level dependent (BOLD) signal) while they watched 14 blocks of images with different valences (positive, negative, or neutral). Each block consisted of one image [47].

The results indicated that ziprasidone treatment led to the activation of several prefrontal regions, which are typically used for cognitive control (such as the anterior cingulate and ventrolateral prefrontal cortex), in patients in response to positive versus negative stimuli. The effect was more obvious when they had to choose negative stimuli rather than positive stimuli. No changes were seen in patients taking other antipsychotics. Additionally, there was an increase in brain volume that was commonly exhibited by healthy controls and patients treated with ziprasidone as a result of the cognitive processing of emotional information. No major changes were seen in the PANSS and CDSS total scores following the ziprasidone treatment, and there were no major fluctuations in weight, glucose, or cortisol levels [47].

### 4.6. Risperidone

We found two relevant studies that tested the efficacy and safety of risperidone. For example, Hori et al. aimed to observe the cognitive effects of oral and injectable risperidone in stable patients with schizophrenia [42]. The participants included in the study were 16 patients with schizophrenia who were initially receiving oral risperidone and were switched to injectable risperidone and 14 participants who continued their treatment with oral risperidone.

Patients were included if their condition was stable in the last 3 months and they were receiving standard treatment for any concomitant medical condition. The exclusion criteria were the following: the presence of other psychiatric conditions other than schizophrenia, a history of epileptic seizures, a suicide attempt, and recent drug/alcohol abuse [42]. All patients completed the Positive and Negative Syndrome Scale (PANSS) [50] and Brief Assessment of Cognition in Schizophrenia (BACS) [83], validated for the Japanese population, initially and at 24 weeks. The results indicate that injectable risperidone is just as efficient and safe as oral risperidone (no major changes were found in PANSS total scores and no major side effects were observed). However, those receiving long-acting injectable risperidone showed greater improvement in verbal memory than those receiving conventional risperidone. These results were attributed to the limited number of participants and the fact that it is a preliminary study [42].

Another study, by Hou et al., aimed to compare the efficacy of three atypical antipsychotics (risperidone, olanzapine, and aripiprazole) in terms of cognitive performance. The participants were 546 patients with acute schizophrenia (first episode) who received risperidone (n = 189), olanzapine (n = 178), and aripiprazole (n = 179) and were evaluated at 6 and 12 months of treatment to see how cognitive performance changes during and after the treatment [46].

Inclusion criteria were the following: a schizophrenia diagnosis established with the Structured Clinical Interview for the DSM-IV Axis I Disorder [84], an age between 16 and 45, psychosis onset after the age of 15, the lack of any previous systematic psychiatric treatment, and a minimum of 3 years of schooling. Exclusion criteria were the following: the presence of various neurological disorders or other relevant medical conditions, prolonged amnesia (>1 h), an intellectual or hearing disability, pregnancy or breastfeeding, previous participation in another clinical trial, and substance abuse or dependence within three months [46]. The instruments used for the clinical and neuropsychological assessment, as well as the safety of the aforementioned antipsychotics were the following: Positive and Negative Syndrome Scale (PANSS) [50], MATRIX Consensus Cognitive Battery (MCCB) [24], and UKU side effect scale [58]. The results indicate that after 6 months, therapy led to significant improvements in most cognitive domains assessed with MCCB [24] (except for verbal learning and memory) in all three groups. Despite improvements in three cognitive domains, visual learning and memory performance dropped back to baseline at 12 months [46].

### 4.7. Haloperidol versus Other Antipsychotics, First- versus Second-Generation Antipsychotics

Harvey et al. aimed to compare the efficacy and safety of blonaserin and haloperidol based on a full analysis dataset instead of a per protocol dataset. Particularly, the researchers examined both blonaserin and haloperidol’s capacity to reduce positive and negative symptoms and improve cognitive functioning [85].

The patients included in the study were aged between 16 and 64 years and had a schizophrenia diagnosis according to ICD-10 criteria. The patients were not eligible for the study if they had the following: a visible state of agitation or lethargy/stupor; a personality disorder; antipsychotic treatment resistance; long-term use of other antipsychotics; and a history of neuroleptic malignant syndromes or water intoxication [85]. The patients were divided 1:1 and were administered between 8 and 24 mg of blonaserin and between 4 and 12 mg of haloperidol, according to the treatment response and tolerability [86,87].

Instruments used for assessing the safety and efficacy of both antipsychotics were the following: Positive and Negative Syndrome Scale (PANSS) [50]; Clinical Global Impressions (CGI) [54]; Brief Psychiatric Rating Scale (BPRS) [88]; and the Drug-Induced Extra-Pyramidal Symptoms Scale (DIEPSS) [89]. The results showed similar efficacy and safety for the two antipsychotics. Blonaserin significantly reduced the frequency of extrapyramidal symptoms. Blonaserin also reduced the severity and frequency of both positive and negative symptoms, the latter effect being attributed to the D3 dopamine receptors and serotonin receptor 5-HT2A [85].

Veselinovic et al. conducted a double-blind, randomized clinical trial, comparing the effectiveness of first-generation antipsychotics (aripiprazole, olanzapine, and quetiapine) with the effectiveness of second-generation antipsychotics (haloperidol and flupentixol) in improving the quality of life and cognitive functioning in schizophrenia [41].

There were 114 participants, of whom 78 were men and 36 were women, aged between 18 and 65 years, diagnosed with a type of schizophrenia based on the ICD-10 criteria. Patients were excluded if they were pregnant or breastfeeding (for women), followed treatment of any kind, were at high risk for suicide, had hypersensitivity/intolerability to any of the study drugs, had a history of neuroleptic malignant syndrome or other chronic somatic diseases, and presented abnormalities in the standard lab tests or electrocardiograms. They were divided into two groups: those receiving first-generation antipsychotics (N = 52) and those receiving second-generation antipsychotics (N = 62).

The drugs were administered twice a day (in the morning and in the evening), one tablet with the active substance and another with the placebo substance [41]. This was necessary to enable the morning administration of aripiprazole and the evening administration of other drugs due to their stronger sedative effect. Depending on the severity of the disorder and particular tolerability, each daily dose varied between two and four tablets per participant. A single pill contained either a placebo substance, 5/7 mg of aripiprazole, 3 mg of flupentixol, 1.5 mg of haloperidol, 5 mg of olanzapine, or 200 mg quetiapine. During the first two weeks of the study, the previous psychopharmacological treatment was ceased, and the patients received one tablet with the active substance every day [41] from then on.

Participants in the study were assessed initially and at 6 and 24 weeks with a battery of neurocognitive tests: Verbaler Lernund Merkfaehigkeitstest (VLMT) to measure verbal memory [90], Regensburger Wortfluessigkeitstest (RWT) to measure verbal fluency [91], and the Letter–Number Span test of working memory performance (LNS) [60]. The Clinical Global Impressions Scale (CGI-S) [54], Positive and Negative Syndrome Scale (PANSS) [50], and Short Form Survey-36 (SF-36) [77] have been used to assess psychopathology as well as quality of life of the patients [41]. The results indicated that the group receiving SGAs showed small-to-moderate improvements in the cognitive functions (especially executive functions and verbal fluency) at both 6 and 24 weeks. However, the group receiving FGAs showed moderately improved executive functions but a decline in verbal fluency at 6 weeks and a decline in executive function, verbal learning/memory, and verbal fluency at 24 weeks.

### 4.8. Antipsychotics and Adjuvants

Boggs et al. analyzed cannabidiol’s efficacy in improving the cognitive functioning of schizophrenia patients. The 39 patients included in this study were aged between 18 and 65 years, had a schizophrenia diagnosis according to DSM-IV-TR criteria, and 3 months of stable antipsychotic treatment. Patients were excluded if they had any past or current DSM-IV-TR Axis 1 [84] disorder that required pharmacological treatment, had a history of substance abuse (excepting nicotine), had a serious medical or neurological disorder, were pregnant/breastfeeding or not willing to use birth control, were enrolled in a weight loss program, had recent exposure to the Hopkins Verbal Learning Test (HVLT) [92], or were taking clozapine, cognitive enhancers, or other experimental medications during the trial. The study participants received either 300 mg cannabidiol/two times a day (N = 20) or a placebo substance (N = 19). The instruments used for the cognitive and clinical assessment were the following: Weschler Adult Intelligence Scale (WAIS) [71], Hopkins Verbal Learning Test (HVLT) [92], Positive and Negative Syndrome Scale (PANSS) [50], Barnes Akathisia Scale (BAS) [80], Simpson–Angus Scale (SAS) [78], and Abnormal Involuntary Movements Scale (AIMS) [54]. The UKU-Side Effect Scale [58] was used for measuring the side effects. The results showed that CBD augmentation did not significantly improve cognitive functioning. On the contrary, cognitive improvements could only be seen in the placebo group. The researchers concluded that this result is due to the learning effect and the regression to the mean [24,38].

Another study that aimed to test the efficacy of cannabidiol was conducted by McGuire et al. and was a randomized controlled trial. Particularly, researchers wanted to investigate the safety and efficacy of cannabidiol as an adjunctive therapy for schizophrenia in patients who were partially responsive to antipsychotic medication. The researchers also observed the effect of cannabidiol on positive and negative symptoms, cognitive functioning, and general functioning [37].

There were initially 88 participants, of which 51 were male and 37 were female, aged between 18 and 65 years and diagnosed with schizophrenia or another psychotic disorder (which was not substance-induced) according to the DSM-IV criteria. Patients were also required to have at least a partial response to antipsychotic medication and a stable treatment over the course of 4 weeks; this treatment continued during the trial. The use of alcohol, cannabis, or other substances was not prohibited, but the patients who had a substance-induced psychotic disorder were excluded. Subsequently, five patients withdrew (two due to adverse effects and three withdrew consent) [37]. Exclusion criteria were the following: a total score <60 on the PANSS scale [50]; the presence of more than one antipsychotic treatment, delirium, dementia, or other disorders that could have put the participant’s life at risk if he had participated in the study; pregnancy and breastfeeding; or planning a pregnancy during the study or within 3 months before its completion [37].

Participants were randomly assigned to the two groups (treatment with 1000 mg CBD per day in addition to the administered antipsychotic or placebo), which were approximately equal (N = 43 in the treatment group and N = 45 in the placebo group) [37] The results indicated that after 6 weeks of treatment, patients in the CBD (cannabidiol) group showed a reduction in positive symptoms, as indicated by the PANSS [50] total score, but also an overall improvement in their general condition, as indicated by the CGI scale [54] score and the clinical assessment performed by the psychiatrist administering the treatment. Also, patients showed an improvement in cognitive functioning, indicated by the total score on BACS [83]. The most frequently reported side effects in the study were gastrointestinal issues, nervous system-related problems (headaches and drowsiness), insomnia, and certain infections. Those side effects were similar for the two groups [37].

### 4.9. Lurasidone

We also found two relevant studies that aimed to test the efficacy of another antipsychotic, namely lurasidone. Thus, Goldman and collaborators wanted to evaluate the efficacy and safety of lurasidone for adolescent patients with acute symptoms of schizophrenia over the course of 6 weeks [33].

There were 380 participants in the study from 14 countries of which only 327 (208 male and 119 female) were eligible following the screenings [33]. The study included adolescents aged 13–17 years; diagnosed with schizophrenia according to DSM-IV-TR criteria; Schedule for Affective Disorders, Schizophrenia for School-age Children (K-SADS-PL) [93]; and who had an exacerbation of acute symptoms (lasting 2 months or more) indicated by a score of ≥70 on the PANSS scale and a score of ≥4 on the Clinical Global Impressions-Severity scale (CGI-S) [54]. Exclusion criteria were the following: the presence of another DSM-IV diagnosis; a history or current diagnosis of intellectual disability or other neurological disorder; alcohol or substance abuse (past 6 months); moderate or severe movement disorders; and a high risk of suicide or injury to self or others. Additionally, patients were excluded if they had a >25% decrease in PANSS total score between baseline and final screening [33].

Before being assigned to the three groups (40/80 mg lurasidone or placebo), participants were taken off any psychotropic medication, with a few minor exceptions where it was absolutely necessary. The study medication, consisting of two tablets, was taken either in the evening with a meal or within thirty minutes after eating. Patients assigned to receive lurasidone 40 mg/day were given that dose on day 1. However, patients assigned to receive lurasidone 80 mg/day were initially given 40 mg/day for three days and 80 mg/day from day 4 onward. Tablet counts from returned blister packs at study visits were used to assess adherence to study medication [33]

The instruments used to assess the effectiveness of the treatment were the Schedule for Affective Disorders, Schizophrenia for School-age Children (K-SADS-PL) [93]; Positive and Negative Syndrome Scale (PANSS) [50]; Clinical Global Impressions (CGI) [54]; and the Pediatric Quality of Life Enjoyment and Satisfaction Questionnaire (PQ-LES-Q) [94]. Safety was assessed based on patient-reported adverse effects and supplemented with the (UKU) Side Effect Rating scale [58]. Psychomotor dysfunctions and extrapyramidal symptoms were assessed using the Simpson–Angus Scale (SAS) [78], the Barnes Akathisia Rating Scale (BARS) [80], and the Abnormal Involuntary Movement Scale (AIMS) [95]. Cognitive functioning was assessed with the Cogstate brief battery (CBB) [96].

The results obtained showed that treatment with lurasidone (regardless of the dose administered) was associated with an improvement in the PANSS [50] total score from week 1 to week 6 for both cohorts (the cohort of patients aged 13–15 and the cohort of patients aged 16–17 years). Moreover, lurasidone-treated patients in both groups showed significantly greater improvement in comparison with the placebo group on the CGI [54] severity subscale and on all of the PANSS subscales. No detrimental effects on cognition were seen; the adverse effects were minimal and similar to those reported in the literature [33].

An interesting experiment carried out by Meltzer and his team aimed to observe whether treatment-resistant schizophrenia patients were much more prone to psychopathological and cognitive improvement after 24 weeks of treatment than only after 6 weeks. The 133 participants in the study were screened for eligibility. In order to determine whether schizophrenia patients were resistant to any treatment, researchers had discussions with their families about the presence and severity of positive symptoms. Medical records of the participants were also reviewed and after that, only 70 patients were eligible for the open trial. The protocol required monitoring of the patients in the first 6 weeks in order to check resistance to the treatment and only patients with a score ≥4 on the PANSS [50] positive subscale were included in the second part of the study [34].

The 6-week open trial of lurasidone 80 mg/day was then followed by a randomized double-blind lurasidone trial which compared the efficacy of either 80 or 240 mg lurasidone/day. Clinical and functional assessments were determined with the Positive and Negative Syndrome Scale (PANSS) [50] and Clinical Global Impressions (CGI)-Severity subscale [54]. For the assessment of neurocognitive functioning, researchers used the Wisconsin Card Sorting Test (WCST) [97], Weschler Intelligence Scale for Children-Revised (WISC-R), Letter Fluency (LF), Category Fluency (CF), Animal Naming (AN) [71], Brown–Peterson Auditory Consonant Trigrams Test (BPT) [98], Digit Symbol Substitution Test (DSST) [99], Rey Auditory Verbal Learning Test (RAVLT) [100], adverse effects were measured using the Abnormal Involuntary Movement Scale (AIMS) [79], Barnes Akathisia Rating Scale (BARS) [80], and Simpson–Angus Scale (SAS) [78].

The results obtained showed that the treatment with 80 mg/d was effective for treatment-resistant schizophrenia patients but required a longer period of treatment (only three patients showed improvements in the PANSS total score in the first 6 weeks). In the second part of the study, lurasidone reduced positive/negative symptoms and improved general psychopathology, as shown by the PANSS total score. Overall functioning measures were more promising between 6 and 24 weeks. Lurasidone significantly improved executive functions and processing speed, both doses being well tolerated by the patients [34].

### 4.10. Olanzapine

A study that investigated the efficacy of olanzapine was carried out by Potkin et al., who wanted to observe the efficacy and safety of a combination of olanzapine and samidorphan compared to olanzapine administered alone in the case of adult patients with an exacerbation of acute symptoms of schizophrenia. The efficacy was determined by changes in PANSS and CGI-Severity subscale scores over the course of 4 weeks [35].

There were 401 participants (244 men and 157 women), of whom only 352 completed the treatment. They were adults, aged between 18 and 70 years, diagnosed with schizophrenia according to DSM-5 and with an exacerbation of acute symptoms (PANSS score ≥80, with scores ≥4 on items 1, 2, 3, and 6; and a CGI score ≥4 on the severity subscale at the initial and final assessment). The patients were randomly assigned to the three groups (20 mg of olanzapine/day; 20/10 mg olanzapine/samidorphan/day; or placebo) and were monitored for 4 weeks. Initially both groups received 10 mg of olanzapine, and then the dose was increased to 20 mg in both groups [35]. Exclusion criteria were the following: significant health problems that could compromise the patients’ safety; a history of diabetes; significant substance abuse in the past 3 months; a positive urine drug test (for either opioids, amphetamine, methamphetamine, phencyclidine, or cocaine); increased risk of suicide; previous exposure to olanzapine, mesoridazine, chlorpromazine, thioridazine, or long-acting injectable antipsychotic medication in the past 6 months; antipsychotic treatment initiated within the past 12 months; less than 1 year since the onset of acute symptoms; clozapine treatment within the past 6 months; history of clozapine use for treatment-resistant or inadequately responding schizophrenia on olanzapine treatment; use of weight loss medications; the use of hypoglycemic agents or statins (either initiated or changed within 3 months); and the use of opioid agonists in the past two weeks [35].

The instruments used were the Positive and Negative Syndrome Scale (PANSS) and Clinical Global Impressions (CGI)-Improvement subscale to assess the effectiveness of the treatment. Assessments regarding the safety of the treatment were conducted using the Abnormal Involuntary Movement Scale (AIMS) [54]; the Barnes Akathisia Rating Scale (BARS) [80]; the Simpson–Angus Scale (SAS) [78]; the Columbia–Suicide Severity Rating Scale (C-SSRS) [101]; clinical laboratory assessments; and electrocardiograms [35]. The results showed that the combination of olanzapine and samidorphan is more effective than a placebo in treating schizophrenia patients with acutely exacerbated symptoms (indicated by PANSS score and CGI-Improvement subscale score) but just as effective as olanzapine alone. The most common reported adverse effects were drowsiness, anxiety, dry mouth, and headache [35].

Zhou et al. aimed to observe the changes in cognitive functioning and symptomatology of schizophrenia patients when the dose of antipsychotic medication (risperidone or olanzapine) was reduced [36]. There were 75 participants, of which 55 were men and 20 were women, aged between 18 and 60 years, with a diagnosis of schizophrenia according to DSM-IV criteria. The stability of symptoms was indicated by a score ≤3 (moderate) on the following PANSS items: P1 (delusion), P2 (conceptual disorganization), P3 (hallucinatory behavior), and P6 (suspicion/persecution) [36].

Patients also had to receive a constant treatment for at least 3 months prior to the study with one of the two antipsychotics (either ≥4 mg/day risperidone or ≥10 mg/day olanzapine), to understand the objectives of the study and to be able to provide informed consent. The exclusion criteria were the following: a treatment with a combination of several antipsychotics (excepting only the combination of ≤50 mg/day clozapine with ≤200 mg/quetiapine, required for patients who had sleep problems), a history or current diagnosis of major neurological disorders or medical conditions, substance abuse, and pregnancy or breastfeeding [36].

The participants were randomly divided into two groups: one receiving treatment in a lower than usual dose and the other receiving maintenance treatment (initial dose). In the first group (those receiving a lower than usual dose), the dose of risperidone or olanzapine was reduced by 25% in the first 4 weeks, then by 50% in the following 12 weeks and maintained like this until the end of the study, which lasted for 52 weeks. In the second group (those receiving maintenance treatment), the initial dose of risperidone or olanzapine was not changed throughout the study. For the safety of all patients, the dose of risperidone administered was not less than 2 mg/day, nor was the dose of olanzapine less than 5 mg/day; those being the standard recommended doses for schizophrenia treatment [36].

The instruments used in the evaluation were the following: Positive and Negative Syndrome Scale (PANSS) [50]; Negative Symptom Assessment-16 (NSA-16) [102]; Rating Scale for Extrapyramidal Side Effects (RSESE) [78]; and the MATRICS Consensus Cognitive Battery (MCCB) [24]. Those instruments were used to assess several cognitive domains including working memory, verbal learning, visual learning, problem solving and social cognition. These measurements were performed at baseline, 12 weeks, 28 weeks and 52 weeks [36].

The results indicated a significant decrease in the negative symptoms (as indicated by Positive and Negative Syndrome Scale (PANSS) [50] negative subscale score and Negative Symptom Assessment 16 scale (NSA-16) score [102]) and a decrease in the extrapyramidal symptoms (as indicated by the Rating Scale for Extrapyramidal Side Effects (RSESE) score). Improvements in processing speed and working memory were also observed (and indicated by the MATRICS Consensus Cognitive Battery (MCCB)) [24] for patients receiving a lower than usual dose of risperidone or olanzapine [36].

**Table 2:** Antipsychotics and their effect on cognition in schizophrenia.

Authors	Antipsychotic/Antipsychotic + Adjuvant with the Highest Effectiveness	Instruments	Main Results
[33]	Lurasidone	K-SADS-PL [93]; PANSS [50]; CGI [54]; PQ-LES-Q [94]; UKU [58]; CGAS [103]; SAS [78]; BARS [80]; AIMS [79]; C-SSRS [101]; CBB [96].	Positive and negative symptoms, the general condition of the patient ↓, psychomotor speed, attention, learning, working memory ↑
[34]		PANSS [50]; CGI [54]; WCST [97]; WISC [104]; LF [105], AN [106] BPT ([98]; DSST [99], RAVLT [100] AIMS [79]; BARS [80]; SAS [78]	Executive function, processing speed ↑, positive and negative symptoms↓, general psychopathology ↓
[35]	Olanzapine	PANSS [50]; CGI [54] C-SSRS [101]; AIMS [79]; BARS [80]; SAS [78]	Positive and negative symptoms, general psychopathology (including psychomotor functions and attention) ↓, general condition of the patient ↑
[36]	Olanzapine + samidorphan	PANSS [50]; NSA-16 [102]; RSESE [78]; MCCB [24]	Negative symptoms ↓, processing speed, working memory, ↑
[37]	Antipsychotic (adjuvant)	WAIS [99]; CGI [54]; PANSS [50]; SANS [49]; BACS [83]; GAF [57]	Positive symptoms ↓, general condition of the patient ↑,
[38]		PANSS [50]; CGI [54]; AIMS [79]; BARS [80]; SAS [78]; UKU [58]; AIMS [79]; HVLT [92]; WAIS [99]	Positive or negative symptoms/general psychopathology -
[39]	Amisulpride/adjuvant	PANSS [50]; CGI [54]; SANS [49]; RBANS [53]; TESS [55]	Positive/negative symptoms ↓, general psychopathology ↓, response time, working memory, visuospatial ability, speech, attention, long-term memory ↑
[40]		PANSS [50]; GAF [57]; CGI [54]; CDSS [75]; UKU [58]	Positive and negative symptoms, general psychopathology ↓
[18]		PANSS [50]; BPRS [88]; CGI [54]; CPT-IP [59]; LNS [60]; SOPT [61]; DRT [62,63]; AVLT [64]; DST [66]; TMT [67]	Visual memory, processing speed ↑, immediate memory ↑, sustained attention, visuo-motor coordination ↑
[41]	Haloperidol Risperidone Quetiapine Aripiprazole	PANSS [50]; CGI [54]; WISC-R [71]; AIMS [79]; MCCB [24]; VLMT [90]; RWT [91]; LNST [60]; CGI [54]; PANSS [50]; SF-36 [77]	Processing speed, cognitive flexibility, verbal memory, working memory ↑;
[42]		PANSS [50]; BACS [83]	Verbal memory, learning ↑
[43]	Clozapine/quetiapine	CPT-IP [59]; SS and LNS of WMS-III [71]; BVMT-R [72]; NAB-Mazes Test [73]; ME of MSCEIT [74]	Attention, working memory, social cognition ↑ related to clozapine
[44]		PANSS [50]; SF-36 [77]; CDSS [75]; SUMD [76]; RFS [107]; SOFAS [51]	Positive symptoms, social cognition ↑
[45]	Paliperidone	MCCB [24]; SANS [49]; PANSS [50] PSP [51]	Processing speed, attention, cognitive functions, social cognition ↑, social functioning and symptomatology -
[46]	Haloperidol, risperidone, olanzapine, aripiprazole	PANSS [50]; MCCB [24]; UKU [58]	Processing speed, executive function, fine motor skills, working memory, attention ↑
[85]	Haloperidol, Blonaserin	PANSS [50]; CGI [54]; BPRS [88]; DIEPSS [89]	Positive/negative symptoms, general psychopathology ↓; Cognitive performance ↑
[47]	Ziprasidone	PANSS [50]; CDSS [75]; BIS [81]; EHI [82]; IAPS [108]	Positive/negative symptoms, general psychopathology -; overall condition of the patient -; response rate for positive stimuli ↑;

↑—increased; ↓—decreased; - remained the same; K-SADS-PL—Affective Disorders, Schizophrenia for School-age Children; PANSS—Positive and Negative Syndrome Scale; CGI—Clinical Global Impressions; CGAS—Children Global Assessment Scale; PQ-LES-Q—Pediatric Quality of Life Enjoyment and Satisfaction Questionnaire; UKU—Udvalg for Kliniske Undersogelser; SAS—Simpson–Angus Scale; BARS—Barnes Akathisia Rating Scale; AIMS—Abnormal Involuntary Movement Scale; CBB—Cogstate brief battery; C-SSRS—Columbia–Suicide Severity Rating Scale; BACS—Brief Assessment of Cognition in Schizophrenia; GAF—Global Assessment of Functioning scale; HVLT—Hopkins Verbal Learning Test; VLMT—Verbaler Lernund Merkfaehigkeitstes; RWT—Regensburger Wortfluessigkeitstes; LNST—Letter–Number Span test; SF-36—Short Form Survey 36; CDSS—Calgary Depression Scale for Schizophrenia; SUMD—Scale to Assess Unawareness of Mental Disorder; NSA-16—Negative Symptom Assessment-16; RSESE—Rating Scale for Extrapyramidal Side Effects; MCCB—MATRICS Consensus Cognitive Battery; BPRS—Brief Psychiatric Rating Scale; EPS—Extrapyramidal Symptoms Scale; PSP—Personal and Social Performance Scale; WCST—Wisconsin Card Sorting Test; WISC-R—Weschler Intelligence Scale for Children-Revised; LF—Letter Fluency;; AN—Animal Naming; BPT—Brown–Peterson Auditory Consonant Trigrams Test; DSST—Digit Symbol Substitution Test; RAVLT—Rey Auditory Verbal Learning Test; SANS—Scale for Assessment of Negative Symptoms; RBANS—Repeatable Battery for the Assessment of Neuropsychological Status; BIS—Barratt Impulsiveness Scale; EHI—Edinburgh Handedness Inventory; TESS—Treatment Emergent Symptom Scale; IAPS—International Affective Picture System; DIEPSS—Drug-Induced Extra-Pyramidal Symptoms Scale.

## 5. Discussion

### 5.1. Efficacy of Antipsychotic Medication in Schizophrenia

After analyzing the relevant literature, we can say that the studies that explored the efficacy of paliperidone or risperidone combined with cognitive training in patients with schizophrenia found no major improvements on cognitive functioning or symptomatology of schizophrenia patients, so we need to carefully consider whether certain antipsychotics combined with cognitive training programs for schizophrenia patients really represent an optimal solution. Still, those treatments may represent a future research direction since they seem to improve social cognition, attention, processing speed, and other executive functions [45].

The results obtained by Zhu et al. show the beneficial effects on cognition of a treatment that combined both clozapine and amisulpride for treating clozapine-treatment-resistant schizophrenia (CTRschizoS). This is due to the possibility that clozapine itself does not reach the required level of D2 receptor blocking, because in order to be effective, the blocking of D2 receptors must reach a percentage of 80% [39,109,110,111,112].

Zhu et al.’s results might have a variety of explanations, including that the degree of D2 receptor occupancy, which could reflect antipsychotic drug plasma concentrations, was associated with vigilance and general neurocognitive functioning in schizophrenia, according to data from the Clinical Antipsychotic Trials of Intervention Effectiveness (CATIE) [113]. Another research study revealed that the inhibition of the D2 receptors caused by risperidone was related to a decreased attention span in schizophrenia patients [114]. Attention, memory, and executive functioning have all been found to be affected as a result of its anticholinergic effects [115,116]. The limited number of participants and the fact that medication adherence was not verified could also have had an impact on the final results.

It is worth mentioning that amisulpride improved both negative and positive symptoms [40,117,118,119]. However, previous research has shown that cannabidiol can also improve cognitive abilities [120,121]. A probable reason is cannabidiol’s affinity for serotonin 5-HT1A receptors, which can compensate for amisulpride’s poor affinity for serotonin receptors [122,123]. Amisulpride is a well-known D2 and D3 receptor antagonist. While the adjuvant effects of cannabidiol are questionable, some studies show that CBD does not significantly improve cognition (not even by itself) [38] while others show the contrary [37]. More studies are needed to examine whether CBD is effective in reducing schizophrenia symptoms [40].

Clozapine also showed promising outcomes, as shown in Park et al.’s study. Park et al. investigated whether the ratio between clozapine and one of its active metabolites, N-Desmethylclozapine (NDMC), can explain cognitive improvement in schizophrenia. Although the results are promising, we must consider the small number of participants in the study (n = 15), as well as the difficulty in controlling factors such as hepatic clearance, systemic metabolism in the gut, smoking, and coffee consumption. Although participants were stable for a long amount of time and the decrease in related positive and negative symptoms was observed, Hui’s team demonstrated the need of following a therapy with clozapine and quetiapine for a longer period of time. The long-term negative effects of these two antipsychotics, such as supersensitivity psychosis and brain volume loss, should also be addressed [43].

Stip et al., 2017 obtained substantial data for frontal lobe activation when ziprasidone was used as a treatment, but their study had a number of limitations, including the size of the experimental group, gender disproportion, and the inability to accurately control the amount of ziprasidone administered; thus, the generalizability of the results is highly questionable. Alzbeta Juven-Wetzler et al. conducted another trial in which ziprasidone medication did not result in any substantial improvement of symptoms in schizophrenia or OCD patients [47,124].

Hori et al. observed the differences between conventional and injectable risperidone. Their results showed that risperidone in smaller doses improved cognition in schizophrenia patients [42]. Veselinovic and his colleagues’ study is also worth mentioning since it emphasized the differences between first- and second-generation antipsychotics, highlighting their generally favorable effect on cognition. We must also consider the multiple confounding factors that may have influenced the results in those studies, such as data coming from a small sample, the lack of statistical adjustments, a high rate of refusal to participate in the study, and a high proportion of withdrawal from study participation, which may have resulted in incomplete or inconclusive results [41].

There are also some drawbacks to Harvey and colleagues’ investigation. First of all, this research’s findings cannot be generalized, mostly because all of the patients were Japanese, and their previous treatment was not mentioned; we cannot say whether the patients’ prior treatment significantly influenced the outcomes. Furthermore, the instruments used to assess the symptomatology of the participants were non-standardized, which could lead to biased or inconsistent results [85].

The study conducted by Meltzer et al. presented some encouraging findings for individuals resistant to any antipsychotic treatment; therapy with lurasidone was demonstrated to be effective in enhancing cognitive functioning. However, the required biomarkers for defining treatment-resistant schizophrenia were not taken into account and there was no control group [34]. It is well-known that lurasidone inhibits dopamine D2 receptors as well as serotonin 5-HT2A and 5-HT7 receptors [125,126]. Lurasidone works as a partial agonist at the serotonin 5-HT1A receptor and has a higher affinity for the 5-HT7 receptor than other atypical antipsychotics.

Moreover, the antipsychotic effect of lurasidone is attributed to the blockage of D2 and 5-HT2A receptors. Lurasidone’s antidepressant properties are assisted by 5-HT7 receptor antagonism and modest 5-HT1A receptor agonism. Lurasidone also inhibits the activation of muscarinic M1, histamine H1, alpha-1, and 2A adrenergic receptors [126]. This level of physical activity lowers the risk of orthostatic hypotension, sleepiness, weight gain, and cognitive blunting induced by other antipsychotic drugs [126,127,128]. Goldman et al. presented a series of benefits of lurasidone as a treatment for young patients with acute psychosis; however, the clinical trial was short (only 6 weeks) and the inclusion and exclusion criteria were rigid, so it is difficult to say how applicable these results are in clinical practice [33].

As for olanzapine, we know that it works as a dopamine D2 receptor antagonist in the mesolimbic pathway, blocking dopamine from acting on the post-synaptic receptor. Olanzapine binds loosely to the receptor and easily dissociates, allowing for normal dopamine neurotransmission. The effect on D2 receptors reduces positive symptoms such as hallucinations, delusions, and abnormal speech, cognition, and behavior. Olanzapine inhibits serotonin 5HT2A receptors in the frontal cortex, which in turn lowers negative symptoms such anhedonia, alogia, avolition, affective flattening, and attention/concentration deficits [129,130]. Potkin and his colleagues’ findings confirm this hypothesis, and that is because D2-type receptors are beneficial in regulating locomotion, attention, sleep, memory, and learning. However, we must keep in mind that the trial was also short (only 4 weeks), and the difference between the two major treatments (olanzapine alone and olanzapine combined with samidorphan) was minimal [35].

The influence of antipsychotics on the cognition and treatment of schizophrenia is further emphasized by two additional studies. These studies demonstrated small beneficial effects of antipsychotics on executive functioning [131] and delayed recall [20].

Clozapine has demonstrated significant beneficial effects on attention and verbal fluency. Additionally, olanzapine has shown a considerable increase in verbal learning and memory, verbal fluency, executive functioning, vigilance, selective attention, and delayed recall [20,131]. When compared to the previously listed drugs, risperidone had fewer negative effects but only moderate effectiveness on working memory, attention, delayed recall, or other executive functions [20,131]. While quetiapine was demonstrated to enhance verbal short-term memory [132], overall cognitive functioning [133,134,135], and reaction time with accurate responses to stimuli [136,137], apipirazole was proven to enhance verbal cognitive ability [138].

A key aspect that must be taken into account is that these results can contribute to a better understanding of the action mechanism of antipsychotic agents on different cognitive domains. More studies like the ones presented, in our opinion, can contribute to the development of new and more efficient treatments that target both schizophrenia’s clinical symptomatology and cognitive impairments, as well as help to better understand the mechanism of action of each antipsychotic on cognitive domains.

It is important to note that while SGAs are thought to be superior in treating patients cognitively, similar benefits could also apply to FGAs. However, from a methodological perspective, all of these studies have limitations. One such example is a meta-analysis, which included nine studies that analyzed the effects of antipsychotics for at least six months. The results obtained show that, when the antipsychotics were taken for eight weeks, there were no significant differences between olanzapine and quetiapine [138,139,140,141,142].

Another such case is lurasidone; while the precise processes behind impact on cognition remain unclear, its strong affinity for 5-HT_7_ receptors may be a significant factor. Behavioral pharmacology studies on rats have shown that lurasidone modulates 5-H_7_ and 5-HT_1A_ receptors to provide anxiolytic, antidepressant, and cognitive beneficial effects. In Vogel’s conflict test, lurasidone, depending on the dose administered, dramatically increased the number of shocks received and anxiolytic behaviors in the rats [126,143].

### 5.2. Limitations

Our comprehensive narrative review regarding the impact of antipsychotic medications on cognitive functioning in schizophrenia may present several limitations.

Firstly, the aim of our review was confined to studies published in the last decade and written exclusively in English. This linguistic and temporal limitation may have inadvertently omitted pertinent studies written in other languages or seminal works preceding our five-year criterion, potentially leading to a selection bias.

The variability in study designs among the included research papers is another significant consideration. The methodologies, sample sizes, and study protocols varied considerably, which introduces challenges in directly comparing and synthesizing the results. Consequently, this heterogeneity could have had an impact on the generalizability and strength of our conclusions regarding the effectiveness of antipsychotics regarding cognitive functioning.

Furthermore, our review may be subjected to publication bias. Studies reporting positive findings are more frequently published than those with negative or inconclusive outcomes. This selective publication could lead to an overestimation of the beneficial effects of antipsychotic medications on cognitive functioning. The quality of the included studies also varied, with some studies exhibiting potential methodological flaws such as small sample sizes, limited duration, or the absence of robust control groups. These limitations can weaken the validity of the conclusions drawn from these studies.

Additionally, the applicability of our findings to the broader schizophrenia population may be limited. The majority of studies we reviewed were randomized controlled trials with rigid inclusion and exclusion criteria, which may not reflect the real-world diversity of the schizophrenia-affected population. Our focus predominantly on the short-term effects of antipsychotic treatment must be considered. The long-term cognitive outcomes and the possible accumulative effects over extended periods remain largely unexplored in our review.

In some instances, the cognitive functioning was assessed using subjective measures, which may not fully encapsulate the true cognitive abilities of individuals with schizophrenia. The inherent subjectivity and potential for bias in these measures could influence the outcomes reported in the studies.

The lack of consensus in the research community regarding the cognitive domains most affected by schizophrenia and its treatment also poses a challenge. This absence of standardization in cognitive assessment can lead to inconsistencies in the evaluation and reporting of cognitive outcomes.

Lastly, various confounding factors, such as baseline cognitive functioning, concomitant medication use, and environmental factors, were not uniformly controlled across the studies. These variables could potentially skew the results and should be taken into account when interpreting our findings.

In light of these limitations, we recommend a cautious interpretation of our review’s conclusions. Future research endeavors in this domain should aim for methodological robustness, diversity in study populations, and a focus on long-term cognitive outcomes to enrich our understanding of the cognitive impacts of antipsychotics in schizophrenia.

## 6. Conclusions

We are already seeing reports regarding the significant and clinically relevant improvements that atypical antipsychotics have on cognitive functioning. There is rising hope that researchers will find a way to reduce the frequency and intensity of schizophrenia symptoms while simultaneously enhancing social and interpersonal functioning. Despite the fact that a number of major studies, including follow-up studies of a naturalistic cohort of patients using a range of relevant outcome measures, are now ongoing, no study has explicitly proved this claim.

It is important to note that the antipsychotics in each study included in this review have positive effects on the cognition and psychopathology of schizophrenia. At the same time, a new trend emerged, and that is treating schizophrenia with different adjuvant substances (such as CBD). However, more studies are needed in order to see whether or not cannabidiol can improve cognition in schizophrenia patients.

## Figures and Tables

**Table 1:** Aims and number of participants for studies between the interaction of antipsychotics and cognition in schizophrenia.

Authors	Aim of the Study	Participants
[33]	Efficacy + safety of lurasidone in acute symptoms of schizophrenia	N = 380 (208 male and 119 female) Age range 13–17 years old
[34]	To see if the treatment was effective (6 vs. 24 weeks)	N = 133
[35]	Efficacy + safety of olanzapine and samidorphan vs. olanzapine	N = 401 (244 men and 157 women) Age range 18–70 years old
[36]	Change in cognition + symptoms via reduced risperidone/olanzapine treatment	N = 75 (55 men and 20 women) Age range 18–60 years old
[37]	Efficacy + safety of antipsychotic and adjuvant (CBD)	N = 88 (51 male and 37 female) Age range 18–65 years old
[38]	Efficacy of an antipsychotic and adjuvant (CBD)	N = 75
[39]	Efficacy + safety of amisulpride on CTRS for cognition and psychopathology	N = 80 Age range 18–65 years old
[40]	Effectiveness of amisulpride vs. aripiprazole vs. olanzapine for the treatment of schizophrenia	N = 144 (51 female and 93 men) Age range >18
[18]	CBD vs. amisulpride for six neurocognitive domains affected by schizophrenia	N = 87
[41]	Effectiveness of FGAs vs. SGAs on cognition and life quality of schizophrenia patients	N = 114 (78 men and 36 women) Age range 18–65 years old
[42]	Cognitive effects of the treatment with injectable vs. oral risperidone for schizophrenia patients	N = 30
[43]	Association between cognitive improvement and clozapine treatment in schizophrenia patients	N = 15 Age range >19 years old
[44]	Long term effects of discontinuing the antipsychotic treatment of schizophrenia patients	N = 178 (80 men and 98 women) Age range 18–65 years old
[45]	Social cognition mediates the relationship between neurocognitive functions and negative symptoms	N = 208
[46]	Risperidone vs. olanzapine vs. aripiprazole in acute schizophrenia at 6/12 months of treatment	N = 546
[47]	Ziprasidone treatment changes in functional and structural brain hemisphere responsible for cognitive functioning	N = 45

CBD—cannabidiol; CTRS—clozapine-resistant treatment-refractory schizophrenia; FGAs—first-generation antipsychotics; SGAs—second-generation antipsychotics.
